# Transcatheter Tricuspid Valve Interventions to Manage Tricuspid Regurgitation: A Narrative Review

**DOI:** 10.31083/RCM39915

**Published:** 2025-08-15

**Authors:** Sara Veneziano, Sabrina Trippoli, Andrea Messori, Vincenzo Calderone, Eugenia Piragine

**Affiliations:** ^1^Department of Pharmacy, University of Pisa, 56126 Pisa, Italy; ^2^HTA Unit, Centro Operativo, Regione Toscana, 50136 Firenze, Italy

**Keywords:** tricuspid regurgitation, valvular disease, transcatheter intervention, medical device, clinical trials

## Abstract

Medical devices for tricuspid regurgitation have emerged as viable treatment options for patients who do not respond to drug therapy or who are unsuitable for open-heart surgery due to high surgical risk. Recently, numerous new medical devices have been proposed and approved for use. Therefore, comprehensive reviews of the literature on the current medical devices for tricuspid regurgitation are necessary. This paper subsequently describes all medical devices used for transcatheter tricuspid valve interventions, providing an updated overview of the current options for managing tricuspid regurgitation, a common valvular heart disease associated with changes in the configuration and function of the tricuspid valve. Over 70 million people worldwide suffer from tricuspid regurgitation, with an estimated mortality rate of 0.51 deaths per 10,000 person-years. However, delays in diagnosis and treatment frequently contribute to disease progression. Meanwhile, the growing health and economic burden of tricuspid regurgitation has led to the urgent need for new therapeutic strategies to overcome the limitations of pharmacological and surgical approaches. In this scenario, transcatheter tricuspid valve interventions represent a promising option for patients with severe tricuspid regurgitation, considered inoperable due to excessive surgical risk. Medical devices designed for these innovative approaches are classified into two main groups: transcatheter tricuspid valve repair and replacement systems. This review presents the technological characteristics of medical devices and the results of studies on their clinical efficacy and safety, thereby supporting the use of transcatheter tricuspid valve repair/replacement systems in clinical practice.

## 1. Introduction 

### 1.1 Epidemiology of Tricuspid Regurgitation

Tricuspid regurgitation (TR) is a common valvular heart disease affecting over 70 million people worldwide [1], with an annual incidence of approximately 200,000 cases in the US and 300,000 in Europe [2]. The estimated mortality rate associated with TR is about 0.51 deaths per 10,000 person-years [3]. The prevalence of TR increases with age and is higher in women than in men, although the reasons for this sex-related difference remain unclear [4].

Chronic untreated TR results in persistent overload and increased wall stress in the right ventricle, leading to a progressive worsening of TR severity and ultimately irreversible myocardial damage [5]. The clinical signs and symptoms of severe TR mirror those of chronic right heart failure (HF), including systemic fluid retention, weakness, dyspnea due to reduced cardiac reserve, and decreased cardiac output that can result in terminal organ failure and progressive damage [6]. Moreover, severe TR is frequently associated with atrial fibrillation [7, 8], which contributes to a further decline in patients’ quality of life, increased incidence of HF, higher mortality rates, and greater healthcare costs [9, 10]. The clinical management of TR imposes a substantial economic burden due to elevated hospital admissions rates, prolonged hospitalization, and other associated healthcare expenses [11]. Therefore, the development of new strategies to slow TR progression and minimize its health and economic impact is urgently needed.

### 1.2 Pathophysiology 

The tricuspid valve (TV), anatomically located between the right atrium and right ventricle, consists of three leaflets of different size situated within an annulus that changes shape—ellipsoidal under normal conditions and more circular and planar during right atrial and/or ventricular diastole [12, 13]. Proper TV function ensures unidirectional blood flow from the right atrium to the right ventricle during diastole and prevents regurgitation during systole. Therefore, any alteration in the valve’s structure or function can result in TR [14]. TR is typically classified into three categories based on etiology: primary TR, secondary TR, and cardiac implantable electronic device (CIED)-related TR [9, 15]. Primary TR, accounting for 5.0–10.0% of all cases, arises from intrinsic leaflet abnormalities due to congenital or acquired conditions [10, 16]. Congenital causes include rare disorders, such as Ebstein’s anomaly [17], while acquired forms may results from infective endocarditis, rheumatic or carcinoid TV disease, drug toxicity, blunt chest trauma, or myxomatous degeneration of the valve leaflets [18, 19]. Secondary or functional TR represents approximately 80.0% of cases and is characterized by inadequate leaflet coaptation. Secondary TR can be further subdivided into atrial secondary TR and ventricular secondary TR [16]. Atrial secondary TR is commonly associated with atrial fibrillation and significant dilation of the annulus and right atrium, leading to impaired leaflet coaptation [20, 21, 22]. Ventricular secondary TR involves right ventricular dilatation and leaflet tethering, typically resulting from elevated pulmonary artery pressure, primary cardiomyopathies, right ventricular ischemia or infraction, or arrhythmias [23, 24]. Notably, ventricular secondary TR is associated with worse prognosis and higher mortality rather than atrial secondary TR [25, 26]. Finally, CIED-related TR, accounting for 10.0–15.0% of all cases [16], is classified separately for epidemiological and therapeutic considerations, although it shares characteristics with both primary and secondary TR [27, 28]. TR occurs in about 19.0–25.0% of patients with permanent pacemakers or implantable cardioverter defibrillators [29, 30], and this rate is expected to rise due to aging populations, increasing CIED implantation, and related complications [31, 32, 33].

### 1.3 Diagnosis and Assessment of TR Severity 

The diagnosis of TR is often incidental, typically discovered during routine imaging in patients with heart disease, since its clinical presentation is non-specific, especially in the early stages. In the presence of suspicious signs and symptoms (e.g., atrial fibrillation, a history of valvular heart disease, pulmonary disorders, mild fatigue, peripheral edema, or dyspepsia), transthoracic echocardiography (TTE) serves as the first-line imaging exam. It is essential to confirm the diagnosis of TR, identify the causes, assess TV anatomy, and determine TR severity [15, 34, 35]. The updated guidelines for echocardiography recommend evaluating TR severity with a multiparametric, hierarchical approach based on a five-class classification system (mild [1+], moderate [2+], severe [3+], massive [4+] and torrential [5+]), in which symptom burden increases progressively with the disease severity [36]. Cardiac magnetic resonance (CMR) and cardiac computed tomography (CCT) are new essential imaging modalities for the comprehensive assessment of TR severity [9, 15], offering quantitative, semi-quantitative and qualitative parameter measurements [16]. Quantitative assessment of TR severity is strongly recommended and typically involves calculating the regurgitant volume and effective regurgitant orifice area (EROA) using the proximal isovelocity surface area (PISA) method [15]. In contrast, qualitative and semiquantitative parameters focus on the structural characteristics of the valvular apparatus and the features of the regurgitant flow jet [37, 38]. Right heart catheterization (RHC) is used to diagnose pulmonary hypertension (PH), distinguish between pre- and post-capillary phenotypes, and obtain a complete evaluation of the hemodynamic profile in patients with TR [39, 40]. According to the European Society of Cardiology and European Association of Cardiovascular Imaging, pre-procedural planning of patients considered for transcatheter interventions should follow an integrated imaging and diagnostic approach. This includes CCT, RHC, and assessment of TR severity with the five-grade classification [15, 33, 35].

### 1.4 Current TR Management Strategies

**Fig. 1.**
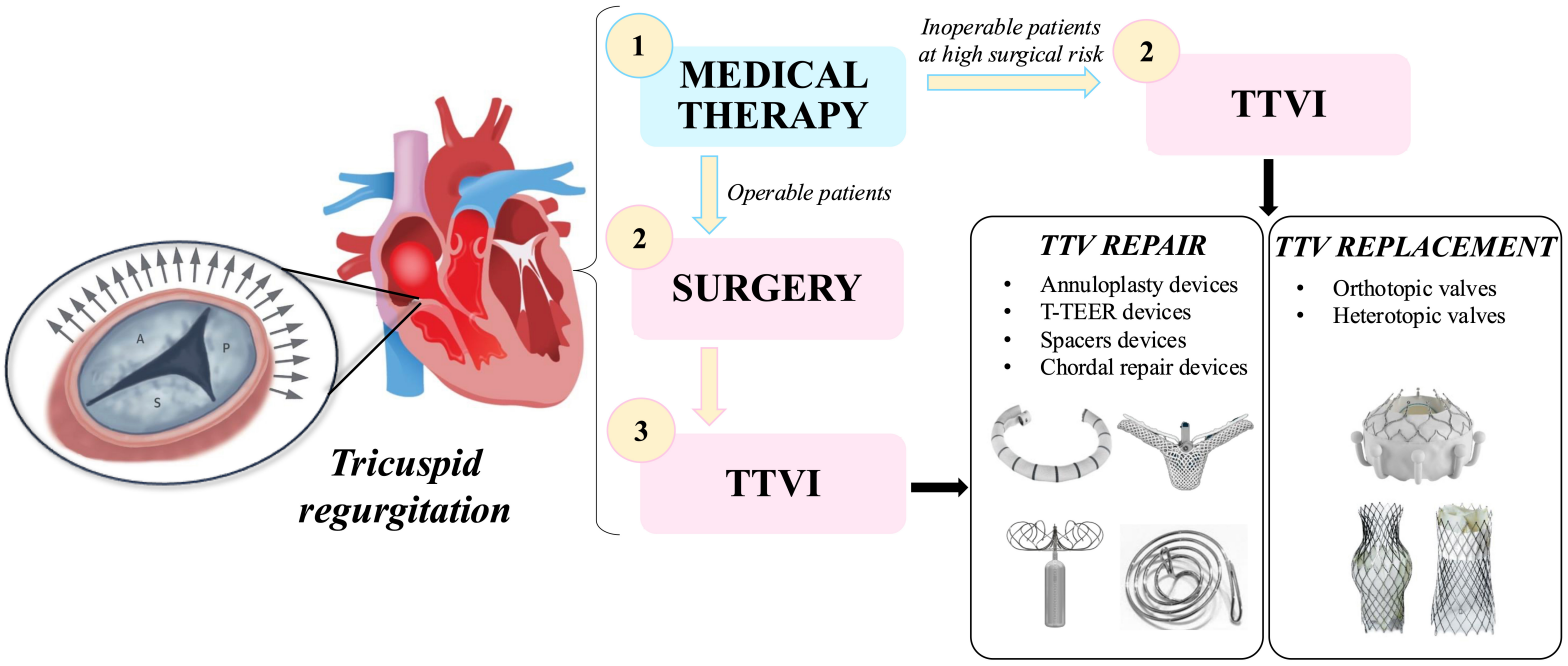
**Schematic overview of tricuspid regurgitation treatment strategies**. List of abbreviations: T-TEER, tricuspid transcatheter edge-to-edge repair; TTV, transcatheter tricuspid valve; TTVI, transcatheter tricuspid valve intervention.

All the diagnostic techniques outlined above are essential for selecting the most appropriate treatment option for each patient with TR [41, 42]. To date, available strategies for the treatment of TR include medical therapy, surgery, and transcatheter interventions. A schematic overview of these management strategies is presented in Fig. 1.

#### 1.4.1 Pharmacotherapy of TR

Medical therapy for patients with TR involves the use of diuretics, most commonly loop diuretics. In more severe cases, mineralocorticoid receptor antagonists may also be employed [33, 43, 44]. In addition, the management of secondary TR requires targeted treatment of underlying conditions, such as PH and atrial fibrillation, that contribute to disease progression [33, 43]. In this regard, one of the most common comorbidities in patients with TR is HF [45, 46], which significantly affects the management and prognosis of the valvular disease. Specifically, HF with preserved ejection fraction (HFpEF) is frequently associated with atrial functional TR (AFTR), while HF with reduced ejection fraction (HFrEF) is common linked to ventricular functional TR (VFTR) [47]. Therefore, it is appropriate to consider HF as an associated condition in the pharmacological treatment of patients with TR, as optimal management of HF can positively influence TR progression and improve clinical outcomes. Besides the “traditional” loop diuretics and mineralocorticoid receptor antagonists, current guidelines recommend the use sodium-glucose co-transporter-2 (SGLT-2) inhibitors in the treatment of HF [44, 48]. A quite recent randomized controlled trial demonstrated that the addition of SGLT-2 inhibitors to optimal medical therapy (i.e., sacubitril-valsartan, beta-blockers, mineralocorticoid receptor antagonists and/or loop diuretics) in patients with HFrEF significantly improved right ventricular function and reduced TR severity compared to the control group (optimal medical therapy) after 3 months of follow-up [49]. These findings suggest that SGLT-2 inhibitors, rather than “traditional” monotherapy, may be more effective in the treatment of TR associated with HF.

#### 1.4.2 Surgery 

Surgery is associated with bad outcomes [50], as several studies have shown high periprocedural and in-hospital mortality rates ranging from 8.2% to 27.6% in patients undergoing TR surgery [51, 52, 53]. Surgical management strategies include TV repair and replacement techniques [10]. However, patients with TR severity grade above “severe” are not suitable candidates for surgical intervention due to the high operative risk and limited expected benefit [33].

Surgical repair is performed by tricuspid annuloplasty and is recommended only for symptomatic patients with isolated severe primary or secondary TR without severe right ventricular dysfunction, as well as in patients with mild to moderate secondary TR and annular dilatation undergoing left-sided valve surgery [33]. This technique involves the implantation of flexible, semi-rigid or rigid rings in the TV annulus to stabilize its diameter [54]. These devices are typically designed with an open structure to minimize the risk of damaging the cardiac conduction system [10], and newer tridimensional (3D) rings have been developed to more accurately replicate the native annular geometry [54]. Direct suture annuloplasty can be performed using the De Vega or Kay techniques, which aim to reduce annular diameter and regurgitant orifice area [10, 55]. The De Vega technique involves annular plication using two continuous suture lines, while the Kay technique achieves bicuspidalization of the TV by obliterating the posterior leaflet [56]. However, suture-based annuloplasty is associated with shorter durability and worse long-term clinical outcomes compared to ring annuloplasty [10]. Finally, the “clover technique” involves joining the free edges of the three leaflets centrally to create a clover-like configuration and enhance coaptation [57].

Surgical valve replacement is indicated in patients with severe annular dilatation or significant leaflet tethering [58]. This procedure typically involves the implantation of either biological or mechanical prosthetic valves via median sternotomy [59]. Recent evidence supports the growing use of minimally invasive surgical techniques due to their procedural advantages and potentially lower perioperative mortality rate [60, 61, 62]. Bioprostheses—composed of porcine valve tissue or bovine pericardium—are preferred in more than 80.0% of patients, due to the lower risk of thromboembolic complications. However, they are less durable compared to mechanical prostheses [63]. Mechanical prostheses, while more durable, carry a higher risk of thromboembolic events, especially in the early postoperative period. Therefore, they are reserved for patients without contraindications to lifelong anticoagulation therapy [59]. Notably, long-term studies have found no significant differences between bioprosthetic and mechanical valves regarding overall survival and freedom from TV-related adverse events over 15 years of follow up [64]. Thus, the choice of prosthesis should be individualized, considering patient’s lifestyle, age, comorbidities, and preferences [33].

#### 1.4.3 Transcatheter Tricuspid Valve Intervention (TTVI)

In recent years, growing clinical awareness of the need for timely and effective management of TR has highlighted the importance of developing new therapeutic strategies to offer tailored interventions and prevent the progression of disease due to delayed diagnosis and treatment [9, 10, 65].

The rapid advancement and favorable clinical outcomes of transcatheter interventions for mitral and aortic valves have encouraged the extension of these innovative techniques to the TV, thus introducing a promising therapeutic option for patients with TR [66]. In this context, TTVI has emerged as a viable treatment for patients with severe symptomatic secondary TR, who are deemed inoperable due to prohibitive surgical risk [9, 10, 14, 65]. However, the development of dedicate TTVI devices is challenged by the complex anatomy of the TV apparatus (e.g., variability in leaflet number and configuration, and its proximity to critical cardiac structures), as well as by the technical demands of percutaneous implantation [67].

To address these challenges, the International TriValve registry was launched in 2016. This global registry includes data from numerous cardiac centers across Europe and North America, documenting all patients undergoing TTVI with any of the currently available devices. Its primary aim is to evaluate patient characteristics and the feasibility safety and procedural outcomes of different TTVI approaches over various follow-up periods [65, 67].

Currently available devices for TTVI can be classified into two main groups based on their mechanism of action: transcatheter tricuspid valve repair systems and transcatheter tricuspid valve replacement systems [66]. The following sections will describe the key design features and implantation procedures of these two types of interventions and provide a comprehensive overview of the clinical efficacy and safety of the respective devices. However, it is important to emphasize that the current evidence remains limited, and further high-quality clinical trials are urgently need to establish the long-term effectiveness and safety profile of TTVI in the management of TR.

## 2. Transcatheter Tricuspid Valve Repair Systems

**Fig. 2.**
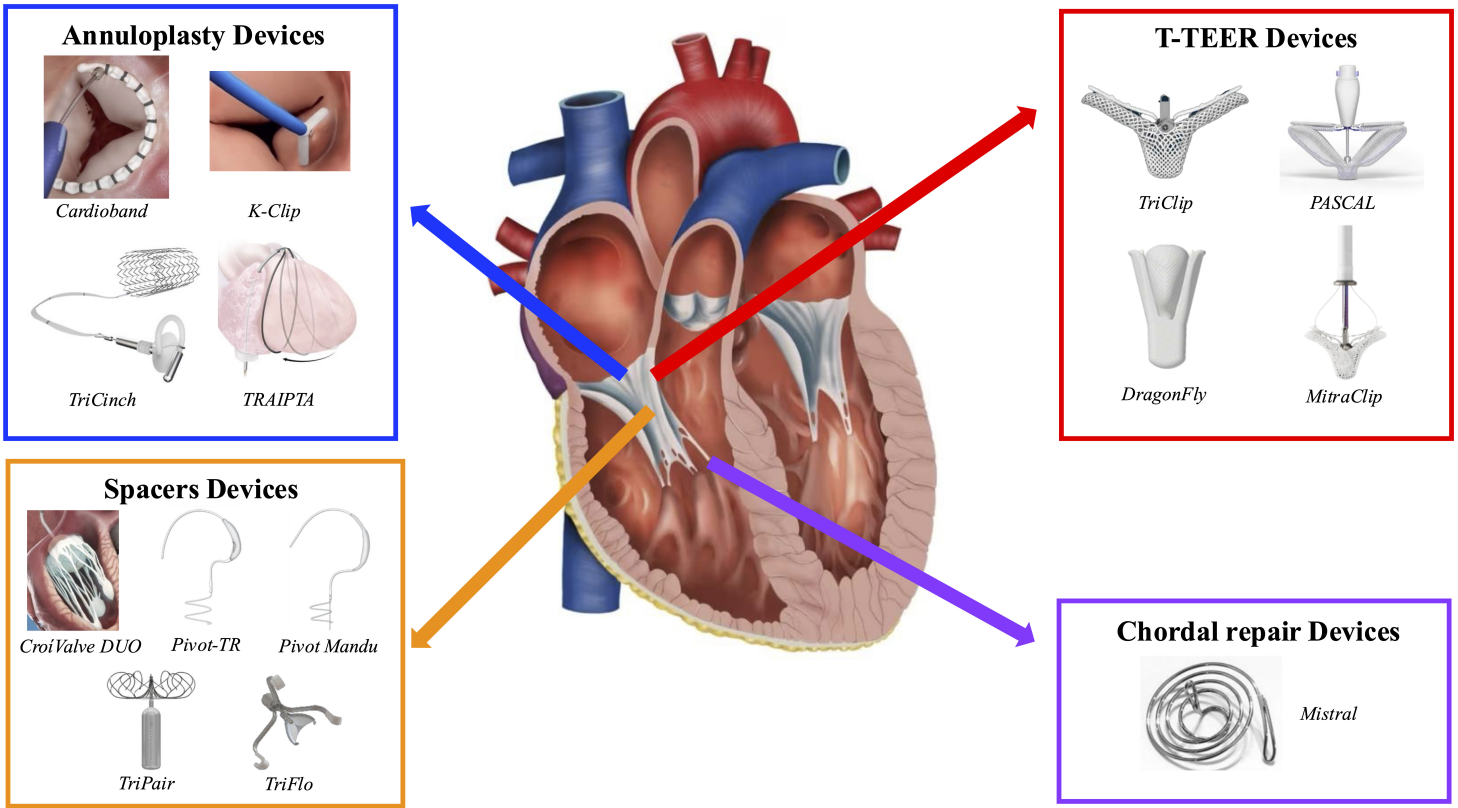
**Currently available transcatheter tricuspid valve repair devices**. List of abbreviations: T-TEER, tricuspid transcatheter edge-to-edge repair; TRAIPTA, transatrial intrapericardial tricuspid annuloplasty; TR, tricuspid regurgitation.

Transcatheter tricuspid valve repair systems can be divided into four categories, based on their specific repair mechanism and anatomical placement [66]. Despite significant advancements in devices for transcatheter repair interventions, a recent cross-sectional study involving 547 patients showed that 41.9% were still considered ineligible for the procedure. The most common reasons for screen failures were anatomical or morphological limitations (58.8%), including excessive dilation of the annulus, right atrium, or right ventricle. Other reasons for exclusion included clinical futility (17.9%), and technical limitations (12.7%) [68]. Fig. 2 provides an overview of the currently available devices developed for transcatheter TV repair. The clinical efficacy of these systems will be discussed in detail in the subsequent sections.

### 2.1 Annuloplasty Devices

Transcatheter annuloplasty is usually performed to reduce excessive pathological annulus dilatation and reproduces the mechanism of the surgical technique of annuloplasty, leading to the same results without altering the anatomical features of the TV apparatus. Despite prolonged procedural times and related issues, several annuloplasty devices have been developed with promising clinical results. In detail, transcatheter annuloplasty systems can be classified into ring and suture-based devices [66, 69]. To date, only the Cardioband system (Edwards Lifesciences, Irvine, CA, USA) has been approved in Europe for the clinical management of TR [70].

#### 2.1.1 Cardioband 

The Cardioband direct ring-based device consists of a polyester fabric covering with radiopaque markers and an internal contraction wire connected to a size-adjusting spool. During the implantation procedure, the device is delivered via an implant catheter through transfemoral venous access to the atrial side of the tricuspid annulus. Is it then secured along the annulus using several anchors and cinched via the contraction wire, thus reshaping the annulus under real-time imaging guidance [9, 69, 71]. In a recent observational study, 58.6% of patients who underwent CCT angiography (CCTA) for pre-procedural screening were deemed unsuitable for Cardioband implantation. The majority of those who failed screening (81.0%) were excluded due to the proximity of the anchors to coronary vessels, defined as less than 7 mm, which contraindicated safe anchor placement [72]. The efficacy of the Cardioband system for TR was first evaluated in the TRI-REPAIR single-arm trial, which included 30 patients with moderate to severe secondary TR. Improvement in TR severity was observed in 76.0% of patients at 30 days, 73.0% at 6 months [70], and 72.0% at 2 years [73]. The ongoing TriBAND post-market study enrolled 61 patients with symptomatic severe or greater secondary TR. At 30 days, 69.0% of patients had TR severity reduced to less than moderate TR [74]. Finally, the U.S. early feasibility study demonstrated an improvement in TR grade from severe or greater to less than moderate in 44.0% of the enrolled patients at 30 days [75], along with high survival and a low rate of HF re-hospitalization at 1 year [76]. A systematic review and meta-analysis confirmed that Cardioband implantation is effective in achieving mechanical improvements, reducing cardiac remodeling, and slowing disease progression in patients with TR. In addition, this study demonstrated the safety profile of Cardioband, showing a low incidence of adverse events and positive long-term outcomes [77]. A retrospective analysis identified a risk of acute kidney injury and hemorrhagic complications (most commonly at the femoral access site, pericardium, and esophagus), particularly in patients with chronic renal dysfunction. However, none of these complications were associated with increased mortality [78]. Finally, a recent retrospective observational study demonstrated that TTVI with Cardioband is effective and safe even in patients with CIEDs. Specifically, intraprocedural success rates were comparable in CIED-carriers and non-CIED carriers, although a trend toward reduced efficacy was observed in patients with lead-associated TR [79].

Effects on Specific Subgroups of Patients

For clinical purposes, analyzing specific patient subgroups is essential to guide cardiologists in selecting the most appropriate medical device. However, very few studies have focused on this important aspect. At this regard, the Cardioband system has been evaluated in patients with different TR phenotypes. In the study conducted by Barbieri and colleagues [80], 30 patients with AFTR and 35 patients with VFTR were enrolled. The annuloplasty device demonstrated comparable efficacy in reducing TR severity by at least two grades in both groups. However, long-term clinical outcomes, including all-cause mortality and hospitalization rates, were not reported. In another recent study, the 1-year survival probability was significantly higher in 62 patients with AFTR compared to 103 patients with non-atrial functional TR undergoing Cardioband implantation (hazard ratio: 0.27; 95% CI: 0.11–0.92) [81]. A greater reduction in TR severity was observed in patients with AFTR over the 1-year follow-up period. In contrast, no significant difference was found in the rate of HF hospitalizations between the two groups (13.3% vs. 22.7%, *p* > 0.05). These controversial findings highlight the need for further studies to validate and expand upon these preliminary results.

#### 2.1.2 K-Clip 

The K-Clip direct annuloplasty system (Huihe Medical Technology, Shanghai, China) consists of two clamp arms and a central corkscrew anchor, which is delivered via jugular venous access and screwed into the anteroposterior commissure of the annulus. Then, the anchor is withdrawn to pull the annular tissue into the open clip arms, that are subsequently closed, thus reducing the annulus diameter [82]. Following positive results from a preclinical study conducted in swine [83], the first clinical trial demonstrated success implantation in all the 15 patients enrolled, with 93.0% showing at least a one-grade improvement in TR severity at 30-day follow-up [82]. Recently, results from a prospective, single-arm clinical trial involving 96 patients with at least severe secondary TR were published. Technical success was achieved in nearly all patients, and, at 1-year follow-up, the majority showed at least a one-grade reduction in TR severity. In addition, a significant reduction in annulus diameter and marked right ventricular reverse remodeling were observed compared to baseline. Survival and freedom from HF hospitalization were 97.8% and 95.1%, respectively [84]. A secondary analysis of 52 patients with TR and concomitant atrial fibrillation revealed significant improvements in functional TR (evidenced by reductions in EROA and tricuspid annulus diameter, as well as by reversal of right heart remodeling) with a favorable short-term prognosis. No deaths or severe adverse events occurred during the 30-day follow-up [85]. Finally, a retrospective study of 81 patients with TR and concomitant right ventricular-pulmonary arterial uncoupling demonstrated that successful K-Clip implantation led to significant improvements in tricuspid annular plane systolic excursion/pulmonary artery systolic pressure (TAPSE/PASP) ratio, quality of life, and TR severity. These findings support and confirm the results of clinical trials in a high-risk patient population [86].

#### 2.1.3 TriCinch 

The TriCinch system (4Tech Cardio Ltd., Galway, Ireland) consists of a stainless-steel corkscrew anchor connected via a Dacron band to a self-expanding nitinol stent, which is delivered through femoral venous access. The anchor is fixed to the antero-posterior portion of the annulus, while the stents are anchored in the inferior vena cava (IVC). The tension created between these components induces remodeling of the annulus [87]. In the PREVENT trial, 18 of 24 enrolled patients with moderate to severe secondary TR underwent successful implantation without procedural complications, and 94.0% showed an improvement in TR severity by at least one grade [10, 88]. Moreover, the new version featuring a coil anchor fixed in the pericardial space was recently implanted for the first time and demonstrated efficacy and safety at one month follow-up [89].

#### 2.1.4 TRAIPTA 

Transatrial intrapericardial tricuspid annuloplasty (TRAIPTA) involves the insertion of a hollow braided nitinol into the pericardial space via a puncture of the right atrial appendage. Then, it is positioned in the atrioventricular sulcus to externally compress the tricuspid annulus and indirectly reduce its diameter [69]. To date, TRAIPTA has only been studied in preclinical studies using swine models with induced secondary TR, demonstrating a promising reduction in annular dilatation [90]. Further device refinements are underway to enable human use; however, the procedure is contraindicated in patients with a history of pericarditis or prior pericardiotomy.

### 2.2 Tricuspid Transcatheter Edge-to-Edge Repair Devices

Tricuspid transcatheter edge-to-edge repair (T-TEER) is the most performed repair procedure, owing to its well-established efficacy and safety. In general, T-TEER involves the implantation of one or more clips that grasps two tricuspid leaflets, bringing them closer together to reduce TR severity. In a retrospective study of 491 patients evaluated for TTVI, unfavorable TV anatomy resulted in screen failure in 32.0% of those considered for T-TEER. Among patients who were excluded, 66.2% had coaptation gap widths greater than 8.5 mm, and 62.4% exhibited TR jets located in the anteroseptal region [91]. To date, the TriClip device (Abbott Vascular, Santa Clara, CA, USA) and the PASCAL system (Edwards Lifesciences, Irvine, CA, USA) have been approved for clinical use in Europe [9, 66].

#### 2.2.1 TriClip 

The TriClip device is the first T-TEER system developed specifically for the treatment of TR [92], and it differs from MitraClip in the guide catheter and delivery system [9]. In the single-arm TRILUMINATE trial, 60.0% of enrolled patients achieved a reduction in TR severity to moderate or less at 30 days [92], increasing to 70.0% at 1-year follow-up [93]. TriClip has demonstrated a favorable safety profile, with low all-cause mortality and hospitalization rates at both 1-year [93] and 2-year follow-ups [94]. Recently published 3-year results further confirmed the long-term efficacy and safety of the device [92]. In the pivotal randomized controlled trial TRILUMINATE, 87.0% of patients treated with TriClip achieved a reduction in TR severity to a moderate or less at 30 days, compared to only 4.8% of patients receiving medical therapy [36]. TriClip was associated with significant improvements in quality of life and a low rate of adverse events at 1-year follow-up [95]. Similar results were observed in the single-arm, prospective TR-Interventional study (TRIS), which demonstrated that the TriClip system improved clinical outcomes in patients with at least severe TR [96]. Finally, the 1-year results of the ongoing bRIGHT observational post-market study confirmed the efficacy and safety of TriClip in a real-world population [97, 98].

#### 2.2.2 PASCAL 

The PASCAL system features two broad paddles with articulating clasps and a central spacer designed to reduce leaflet stress by filling the regurgitant orifice [99]. In the single-arm CLASP TR trial, 85.0% of patients with successful device implantation experienced at least one-grade improvement in TR severity at 30-day follow-up [100]. At 1-year, all patients demonstrated a reduction in TR severity of at least one grade, along with high survival rates attributed to low all-cause mortality and HF hospitalization [101]. The ongoing CLASP II TR randomized controlled trial is designed to evaluate the long-term efficacy and safety of the PASCAL system compared with medical therapy over a 5-year follow-up [102]. The efficacy and safety of the device have also been confirmed in the real-world setting [103]. Additionally, a large observational study involving over 1000 patients showed that 83.0% experienced TR severity reduction at 1 year, accompanied by significant clinical improvement. Notably, the newer PASCAL Precision system demonstrated advantages over the first-generation device, including shorter procedure times, greater TR reduction, and higher rates of clinical success [104].

#### 2.2.3 DragonFly 

The DragonFly device (Valgen Medical, Hong Kong, China), which has demonstrated efficacy and safety for the treatment of mitral regurgitation (MR) [105], has also been investigated in a patient with TR. At the 1-month follow-up, the patient experienced no serious or severe adverse events and exhibited only mild residual TR [106].

#### 2.2.4 MitraClip 

Although originally developed for the treatment of MR, MitraClip (Abbott Vascular, Santa Clara, CA, USA) was widely used off-label in early T-TEER procedures before the advent of devices specifically designed for the TV [2]. Studies evaluating the efficacy of MitraClip in patients with TR demonstrated a significant reduction in TR severity of at least one grade in 90.0% of patients at 6 months post-implantation [107], with this improvement maintained in the majority of patients at 1-year follow-up [108].

#### 2.2.5 Effects on Specific Subgroups of Patients

As previously reported for Cardioband, data from the TriValve registry demonstrated that patients with AFTR (n = 65) had a significantly better 1-year survival compared to those with VFTR (n = 233), following T-TEER device implantation (MitraClip was used in 95.0% of cases). Additionally, patients with left ventricular ejection fraction (LVEF) ≥50.0% or atrial fibrillation showed a higher, albeit not statistically significant, probability of survival than those with LVEF <50.0% or no atrial fibrillation [109]. These findings were corroborated by another observational study from the European Registry of Transcatheter Repair for Tricuspid Regurgitation (EuroTR), which included 641 patients with TR undergoing T-TEER, followed over a 2-year period. Results demonstrated that patients with atrial secondary TR experienced greater TR reduction, lower symptomatic burden, and significantly higher 2-year survival free from HF hospitalization compared to those with non-atrial secondary TR (66.3% vs. 47.5%) [110]. Finally, Scheffler and colleagues [111] analyzed 136 patients with severe TR treated with either PASCAL (86.0%) or TriClip (14.0%). Despite comparable success in TR reduction, patients with AFTR had a lower 1-year probability of all-cause death and re-hospitalization for decompensated HF compared to those with VFTR [111]. Taken together, these findings suggest that clinical outcomes following T-TEER may vary depending on TR phenotype, with ventricular TR potentially serving as a predictor of worse prognosis.

### 2.3 Spacer Devices

All spacer devices feature a central spacer connected to an anchoring system. This unique configuration reduces the regurgitant orifice area without impacting the annular structures, regardless of annular size and coaptation gap. However, clinical data remain limited, and further studies are needed to confirm their efficacy and safety [69, 112].

#### 2.3.1 CroíValve DUO, Pivot-TR, and Pivot-Mandu 

The CroíValve DUO (CroíValve, Dublin, Ireland) consists of a spacer coupled with a valve apparatus that is anchored to the superior vena cava (SVC) via a stent system delivered through transjugular venous access. The spacer enhances leaflets coaptation and reduces the regurgitant orifice area, while the valve promotes proper diastolic filling flow [69]. Preclinical studies in swine demonstrated significant improvements in TR severity at 30 days [113]. These findings were further supported by a successful implantation in a patient with massive TR and a right ventricular pacemaker lead, resulting in a significant reduction of TR severity from massive to mild at 90 days post-procedure [114].

The Pivot-TR (Tau-PNU Medical Co, Yangsan, South Korea) features a spacer with an open cavity design and a C-shaped pivot axis composed of a long “elephant nose” and a spiral anchor, which is non-traumatically secured to the IVC via transfemoral venous access [115]. The spacer is designed to vertically traverse the valve apparatus, effectively filling the regurgitant orifice. To date, Pivot-TR has been evaluated only in swine with induced TR, demonstrating promising procedural feasibility and significant reduction in TR severity compared to baseline [115]. To address the risk of clot formation due to stagnant blood in the cavities of the Pivot-TR device, the Pivot Mandu (TAU MEDICAL Inc, Yangsan, South Korea) was developed with a leak-tight, adjustable 3D balloon spacer. A preclinical study in pigs with induced TR showed a reduction in TR by more than one grade immediately after implantation without any thrombotic complications [112]. Translational studies are anticipated to confirm these promising results in human patients.

#### 2.3.2 TriPair and TriFlo

The TriPair (Coramaze Technologies, Tikva, Israel) consists of a central flexible column and a crown designed for atraumatic anchoring in the right atrium via transfemoral venous access.

TriFlo (TriFlo Cardiovascular) is a new device featuring a three-anchor system with a central “mini-valve”, which mimics the design and function of the CroíValve DUO system [69]. To date, no preclinical or clinical data are available regarding the efficacy of either the TriPair and TriFlo devices [69, 116].

### 2.4 Chordal Repair Devices

The Mistral device (Mitralix, Yok’neam, Israel) is currently the only device classified as a chordal repair system due to its unique mechanism of action, which ultimately improves leaflets approximation. It consists of a single spiral-shaped nitinol wire delivered via a specific catheter. The device is positioned between the papillary muscle and the leaflet tips inside the right ventricle through femoral venous access. Then, it is carefully rotated clockwise to nontraumatically grasp the *chordae tendineae*, forming a characteristic “flower-bouquet” structure that approximates the *chordae* and leaflets [9, 117]. In first-in-human studies, 17 patients were successfully implanted with Mistral, demonstrating significant improvements in efficacy outcomes without major device-related complications at 30-day and 6-month follow-ups [117, 118]. At 1 year, all patients showed at least a one-grade reduction in TR severity and favorable cardiac remodeling [119].

The key experimental and clinical findings of currently available transcatheter tricuspid valve repair devices, including those approved by the U.S. Food and Drug Administration (FDA) or marked with the Conformité Européenne (CE) certification, are summarized in Table 1 (Ref. [36, 70, 71, 73, 74, 75, 76, 77, 78, 79, 80, 82, 84, 85, 86, 90, 92, 93, 94, 95, 96, 97, 98, 100, 101, 103, 104, 106, 107, 108, 112, 113, 114, 115, 117, 118, 119, 120, 121, 122, 123]).

**Table 1.** **Summary of the key findings from preclinical and clinical studies on currently available transcatheter tricuspid valve repair devices**.

First author, year		Device name	FDA approved and/or CE-marked	Operational mechanism	Main results	Maximum follow-up
Preclinical studies						
	Rogers, 2015 [90]	TRAIPTA	No	Annuloplasty	↓ Annular dilatation in swine	9.7 days
	Curio, 2019 [113]	CroíValve DUO	No	Spacer	↓ TR severity in swine	30 days
	Chon, 2022 [115]	Pivot-TR	No	Spacer	↓ TR severity in swine	6 months
	Chon, 2023 [112]	Pivot Mandu	No	Spacer	↓ TR severity ≥1 grade in pigs	6 months
Clinical studies						
	Barbieri, 2023* [80]	Cardioband	Yes	Annuloplasty	↓ TR severity ≤ moderate	Discharge
	Davidson, 2021* [75]	Cardioband	Yes	Annuloplasty	↓ TR severity ≤ moderate	30 days
	Gerçek, 2021* [120]	Cardioband	Yes	Annuloplasty	↓ TR severity ≤ moderate	Discharge
	Gietzen, 2025 [78]	Cardioband	Yes	Annuloplasty	↑ Risk of acute kidney injury	3 months
					↑ Hemorrhagic complications	
					No ↑ mortality	
	Gray, 2022* [76]	Cardioband	Yes	Annuloplasty	↑ Survival	1 year
					↓ HF re-hospitalization	
	Körber, 2021* [71]	Cardioband	Yes	Annuloplasty	↓ TR severity ≤ moderate	6 months
	Mattig, 2023* [121]	Cardioband	Yes	Annuloplasty	↓ TR severity ≤ moderate	Discharge
	Nickenig, 2019* [70]	Cardioband	Yes	Annuloplasty	↓ TR severity	6 months
	Nickenig, 2021* [73]	Cardioband	Yes	Annuloplasty	↓ TR severity	2 years
	Nickenig, 2021* [74]	Cardioband	Yes	Annuloplasty	↓ TR severity ≤ moderate	30 days
					↑ Survival	
	Ochs, 2024* [122]	Cardioband	Yes	Annuloplasty	↓ TR severity	6 months
					↑ Survival	
	Pardo Sanz, 2022* [123]	Cardioband	Yes	Annuloplasty	↓ TR severity	279 ± 246 days
					↑ Survival	
	Wrobel, 2025 [79]	Cardioband	Yes	Annuloplasty	↓ TR severity	30 days
					Intraprocedural success in patients with CIEDs	
	Lin, 2025 [85]	K-Clip	No	Annuloplasty	↓ EROA	30 days
					↓ Annulus diameter	
					Right heart reverse remodeling	
	Lin, 2025 [86]	K-Clip	No	Annuloplasty	↓ TAPSE/PASP	6 months
					↑ Quality of life	
					↓TR severity	
	Zhang, 2023 [82]	K-Clip	No	Annuloplasty	↓ TR severity	30 days
					by at least 1 grade	
	Zhang, 2025 [84]	K-Clip	No	Annuloplasty	↓ TR severity	1 year
					by at least 1 grade	
					↓ Annulus diameter	
					Right ventricular reverse remodeling	
					↑ Survival	
					↑ Freedom from HF hospitalization	
	Carpenito, 2025 [96]	TriClip	Yes	T-TEER	↓ TR severity	1 year
					Right ventricular reverse remodeling	
	Lurz, 2021 [93]	TriClip	Yes	T-TEER	↓ TR severity ≤ moderate	1 year
					↓ All-cause mortality	
					↓ All-cause hospitalization	
	Lurz, 2023 [97]	TriClip	Yes	T-TEER	↓ TR severity ≤ moderate	30 days
	Lurz, 2024 [98]	TriClip	Yes	T-TEER	↓ TR severity ≤ moderate	1 year
					↓ Mortality	
	Nickenig, 2024 [92]	TriClip	Yes	T-TEER	↓ TR severity ≤ moderate	3 years
					↓ HF hospitalization	
	Baldus, 2022 [103]	PASCAL	Yes	T-TEER	↓ TR severity ≤ moderate	30 days
					↓ Major adverse events	
	Wild, 2025 [104]	PASCAL Precision system	Yes	T-TEER	↓ TR severity	30 days
	Sorajja, 2023 [36]	TriClip	Yes	T-TEER	↓ TR severity ≤ moderate	30 days
	Tang, 2025 [95]	TriClip	Yes	T-TEER	↑ Quality of life ↓ frequency of adverse events	1 year
	von Bardeleben, 2023 [94]	TriClip	Yes	T-TEER	↓ TR severity	2 years
					↓ All-cause mortality	
					↓ All-cause hospitalization	
	Kodali, 2021 [100]	PASCAL	Yes	T-TEER	↓ TR severity by at least 1 grade	30 days
	Kodali, 2023 [101]	PASCAL	Yes	T-TEER	↓ All-cause mortality	1 year
					↓ All-cause hospitalization	
	Ren, 2025 [106]	DragonFly	No	T-TEER	↓ TR severity to mild	1 month
	Orban, 2018 [107]	MitraClip	Off-label use	T-TEER	↓ TR severity by at least 1 grade	6 months
	Mehr, 2019 [108]	MitraClip	Off-label use	T-TEER	↓ TR severity by at least 1 grade	1 year
	Peszek-Przybyła, 2024 [114]	CroíValve DUO	No	Spacer	↓ TR severity to mild	90 days
	Planer, 2020 [117]	Mistral	No	Chordal repair	↓ TR severity by at least 1 grade	30 days
	Danenberg, 2023 [118]	Mistral	No	Chordal repair	↓ TR severity	6 months
	Piayda, 2023 [119]	Mistral	No	Chordal repair	↓ TR severity by at least 1 grade	1 year

* Included in the systematic review and meta-analysis by Piragine *et al*. (2024) [77]. List of abbreviations: CIEDs, cardiac implantable electronic devices; HF, heart failure; TAPSE/PASP, tricuspid annular plane systolic excursion/pulmonary artery systolic pressure ratio; TR, tricuspid regurgitation; EROA, effective regurgitant orifice area; FDA, U.S. Food and Drug Administration. Symbols: ↑ increased; ↓ decreased.

## 3. Transcatheter Tricuspid Valve Replacement Systems

Transcatheter tricuspid valve replacement systems are designed to mimic the characteristics of surgical valve replacement, while avoiding its associated risks, and aim to completely nihilate TR.

**Fig. 3.**
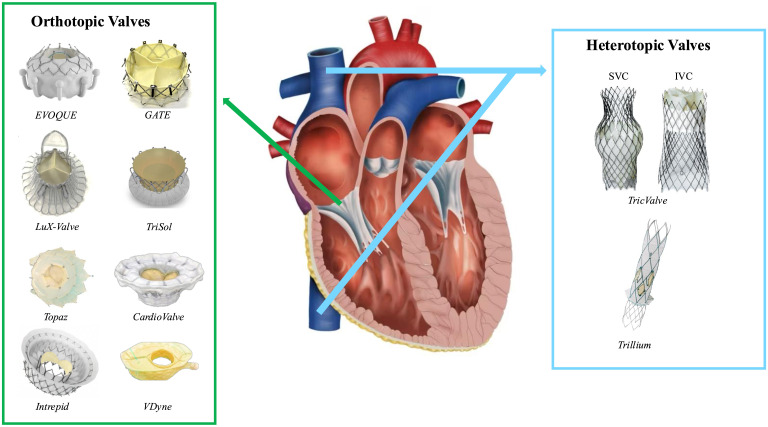
**Currently available transcatheter tricuspid valve replacement devices**. List of abbreviations: IVC, inferior vena cava; SVC, superior vena cava.

These replacement devices overcome the morphological limitations of repair systems and can be implanted even in patients with CIED-related TR. Transcatheter tricuspid valve replacement systems are further classified as orthotopic or heterotopic valves, based on their anatomical positioning [55, 69]. In a retrospective study of 327 patients who underwent pre-procedural CCT, transcatheter tricuspid valve replacement was deemed anatomically and clinically unsuitable in 62.7% of patients. The primary reasons for ineligibility included a tricuspid annulus diameter greater than 53 mm (65.4%), which exceeds the maximum size supported by currently available protheses, and severe right ventricular dysfunction (27.3%). Furthermore, patients presenting with severe leaflet prolapse or excessive flail were excluded due to the high risk of valve fixation failure [91]. To date, the EVOQUE valve (Edwards Lifesciences, Irvine, CA, USA) and the TricValve (P & F Products & Features Vertriebs GmbH, Vienna, Austria) have been approved for clinical use in Europe [124, 125]. Fig. 3 provides an overview of the available devices for transcatheter tricuspid valve replacement.

### 3.1 Orthotopic Valves

#### 3.1.1 EVOQUE

The EVOQUE valve consists of three-leaflet derived from bovine pericardium mounted on a self-expanding nitinol frame equipped with nine anchors and an intra-annular sealing skirt. The valve is delivered via transfemoral venous access and positioned between the leaflets and papillary muscles heads, expanding at annular level [69, 126]. In a compassionate-use study that involved 25 patients with severe secondary TR, procedural success was achieved in 92.0%, with 96.0% of patients experiencing a reduction in TR grade to moderate or less at 30 days [126]. In the single-arm TRISCEND trial, 98.0% of patients achieved improvement in TR grade to mild or lower, accompanied by high survival rates and low hospitalization at 30 days [127]. At one year, 97.6% of patients showed a reduction in TR grade to mild or lower, and the high survival and low hospitalization rates demonstrated the safety of the medical device [128]. The ongoing pivotal TRISCEND II trial is the first randomized controlled trial evaluating the efficacy and safety of the EVOQUE valve compared with medical therapy [129]. At one year, 99.1% of patients receiving the EVOQUE valve had TR severity less than moderate, compared to only 16.1% of those treated with medical therapy [130]. Furthermore, EVOQUE valve implantation was associated with significant improvements in patients’ quality of life, although longer follow-up periods are required to confirm the long-term efficacy and safety of this device [130, 131]. In fact, this randomized trial has some limitations, which were very recently highlighted in a letter to the editor. Specifically, the primary outcome was based on “wins” derived from soft endpoints, such as New York Heart Association (NYHA) class and quality-of-life questionnaires, both of which are highly susceptible to placebo effects. Furthermore, the most objective indicator of clinical improvement was a modest increase of 30 meters in the six-minute walk distance [132].

#### 3.1.2 GATE 

The first transcatheter tricuspid valve replacement device was the GATE system (NaviGate Cardiac Structures Inc., Lake Forest, CA, USA), which consists of a three-leaflet valve derived from equine pericardium mounted on a self-expanding nitinol conical stent, available in four diameter sizes [133]. Anchoring to the TV is achieved by grasping the leaflets using 12 tines on the ventricular side and 12 winglets on the atrial side [134]. The first human implantation was performed via a transatrial surgical approach following a mini-thoracotomy, demonstrating procedural feasibility and improvements in TR severity [133, 135]. In a recent compassionate-use study, 30 patients with severe or greater secondary TR underwent device implantation via direct transatrial approach or transjugular venous access, with a procedural success rate of 87.0%. All patients showed a reduction in TR severity of more than one grade at discharge. However, four patients died during a mean follow-up of 127 days. During the implantation procedure, second- and third-degree atrio-ventricular (AV) block occurred in three patients (10.0%), two of whom subsequently required permanent pacemaker implantation [136].

#### 3.1.3 LuX-Valve and LuX-Valve Plus 

The LuX-Valve (Jenscare Biotechnology, Ningbo, China) is composed of a three-leaflet bovine pericardial valve integrated into an external self-expanding nitinol stent, featuring an atrial disc, two polytetrafluoroethylene-covered graspers, and tongue-shaped anchor for intraventricular septal fixation. The device is delivered into via transatrial access following a mini-thoracotomy, with deployment involving grasping of the anterior leaflet and positioning of the atrial disc [137, 138]. In the first-in-human study, the LuX-Valve was successfully implanted in 12 patients with severe TR, with 90.9% achieving mild or no TR reduction at 30-day follow-up [137]. Despite one post-procedural death, 85.7% of the remaining 14 patients maintained mild or no TR at 1-year follow-up, confirming the efficacy and safety of this device [138]. In the single-arm, prospective TRAVEL study, 126 patients with severe TR underwent Lux-Valve implantation without intraoperative complications. At the 1-year follow-up, nearly all patients demonstrated TR reduction to mild or less, indicating sustained therapeutic benefit. New-onset of AV-block occurred in only two patients, and both required permanent pacemaker implantation, although these events were not directly procedure-related [139].

Recently, the LuX-Valve Plus system was developed with a delivery catheter designed for jugular venous access. In its first-in-human experience, the device was implanted in 10 patients with severe TR, all of whom achieved TR reduction to mild or less at 30 days. Two days post-procedure, one patient developed third-degree AV-block and required pacemaker implantation [140]. Worthy to note, a comparative study involving 28 patients with severe TR randomized participants 1:1 to receive either the Lux-Valve or the Lux-Valve Plus. The Lux-Valve Plus group experienced significantly lower intraoperative bleeding and shorter hospital stays. However, both devices demonstrated comparable efficacy in TR reduction [141].

#### 3.1.4 Trisol

TriSol (TriSol Medical, Yokneam, Israel) is a transcatheter valve specifically designed for the tricuspid position [9]. It features a bovine pericardial monoleaflet mounted within a self-expanding conical nitinol frame, equipped with six circumferential fixation arms that anchor the device between the native leaflets and surrounding tissue. The unique monoleaflet is supported by two commissures that enable dynamic opening during diastole and closing during systole. The first-in-human implantation was successfully performed via jugular vein access, resulting in a significant reduction in TR at the 2-week follow-up [142]. A first single-arm trial is currently underway to assess the efficacy and safety of the TriSol valve [143].

#### 3.1.5 Topaz

Topaz (TRiCares GmbH, Aschheim, Germany) is a transcatheter valve composed of a three-leaflet bovine pericardium valve mounted within a dual-stent nitinol structure with distinct functional components. The outer stent provides anchorage to the native TV, while the rigid inner stent supports the valve, preserving its structural integrity. The device is implanted via transfemoral access, with sequential deployment of the ventricular component followed by the atrial portion. The first-in-human implantation was successfully performed in two patients with massive or torrential secondary TR. Both patients exhibited complete TR resolution and no complications at 3-month follow-up [144]. The ongoing TRICURE trial is currently assessing the safety and performance of the Topaz valve [145].

#### 3.1.6 CardioValve 

CardioValve (Cardiovalve Ltd., Tel Aviv, Israel) is a three-leaflet valve made of bovine pericardium, supported by a dual (atrial and ventricular) self-expanding nitinol frame featuring 24 graspers. After the valve is atraumatically anchored to the native leaflets, fixation is further enhanced by the Dacron-covered flange of the atrial structure. CardioValve is available in four diameters and is designed for use in both the mitral and tricuspid positions [146, 147]. Transfemoral implantation of the device was successfully performed in two patients with torrential secondary TR, who showed complete TR resolution at 3-month follow-up [147].

#### 3.1.7 Intrepid

The intrepid system (Medtronic Inc., Minneapolis, MN, USA), originally developed for the treatment of MR, comprises a three-leaflet valve made from bovine pericardium and a circular stent with an outer frame that enables its distinctive atrial-ventricular anchoring mechanism. To date, this valve system has been implanted via transfemoral venous access in a single patient with severe TR, demonstrating promising outcomes at 30-day follow-up [69]. An ongoing trial is currently enrolling patients to assess the early feasibility of this approach [148].

#### 3.1.8 VDyne

The VDyne valve (VDyne Inc., Maple Grove, MN, USA) is currently under investigation, with no preclinical or clinical results published to date. A single-arm trial is recruiting patients with moderate to severe TR to assess the efficacy and safety of the VDyne valve [149].

### 3.2 Heterotopic Valves

Heterotopic valves are devices specifically designed to reduce the TR-induced blood backflow into the *venae cavae*. Caval valve implantation (CAVI) serves as a palliative treatment option for patients who are not candidates for other transcatheter techniques or surgical replacement due to factors such as excessive annular dilatation, large coaptation gaps, or carcinoid heart disease [69, 150].

#### 3.2.1 TricValve

TricValve consists of two self-expanding nitinol stents, each housing a bovine pericardium valve designed for deployment in the SVC or IVC [151]. In detail, the SVC device features a central belly to prevent dislodgement, a lower covered portion to reduce paravalvular leakage, and an uncovered upper crown that ensures proper accommodation without obstructing blood flow. Conversely, the IVC device has a short lower section to avoid hepatic vein obstruction and a higher upper radial force to support the attachment. Both valves use the same delivery system via transfemoral access and are implanted from above with controlled downward traction until fully deployed. The SVC valve placement is further supported by a rigid wire introduced through the right subclavian or internal jugular vein [69, 125]. TricValve received European approval based on the efficacy and safety outcomes of the TRICUS EURO trial. In this single-arm trial, 35 patients with severe symptomatic TR were eligible for CAVI, achieving a 94.0% procedural success rate. At 6 months, most patients showed significant improvements in quality of life and HF symptoms, with only three deaths reported [125]. Furthermore, the 1-year results from the combined TRICUS EURO and TRICUS cohorts further confirmed the efficacy and safety of TricValve [152]. Finally, the TRICAV trial has been designed to evaluate the long-term safety and efficacy of TricValve [153].

TricValve is currently the only dedicated device for heterotopic TV replacement, such as CAVI, which represents an additional treatment option for patients with severe TR who are not candidates for direct valve repair or replacement. These include patients with previously failed transcatheter repair procedures due to partial leaflet detachments, as well as those with an insufficient response to initial therapy. The rationale behind CAVI is that preventing venous backflow within the *venae cavae* could reduce symptoms of congestion and improve renal and liver perfusion, potentially leading to positive right ventricular remodeling and a reduction in TR severity. Importantly, the heterotopic position of the device may also allow for a second intervention through the valved stent [154, 155, 156].

This bailout strategy has been successfully performed in a 71-year-old female patient with multivalvular rheumatic heart disease by implantation of the TricValve system. However, the patient experienced a progressive deterioration of right ventricular function and worsening symptoms [156]. In another case, an 86-year-old male patient with peripheral edema and worsening dyspnea due to TR and single leaflet device attachment underwent CAVI with the TricValve device. At follow-up, the patient demonstrated adequate caval valve function with no signs of regurgitation, while right ventricular function remained unchanged compared to pre-implantation [155]. Future studies with longer follow-up are needed to clarify the potential role of heterotopic TV replacement in TTVI and to investigate the associated risks, following a first reported case of thrombosis in an 80-year-old female patient who underwent heterotopic TricValve implantation [157]. In addition, further investigation is necessary regarding the off-label use of other devices in this context [158, 159].

#### 3.2.2 Trillium

The Trillium device (Innoventric Ltd., Ness-Ziona, Israel) consists of a metallic stent with an exposed upper crown and a lower sealing skirt, which are deployed into the SVC and IVC via transfemoral venous access. The atrial portion is circumferentially fenestrated by with multiple valves that facilitate blood flow from the venous system to the right atrium, thus preventing reflux [160, 161]. To date, an ongoing clinical trial is evaluating the safety and performance of Trillium in 20 patients [162], and an early feasibility trial is planned to begin enrollment soon [163].

The key experimental and clinical findings of currently available transcatheter tricuspid valve replacement devices are summarized in Table 2 (Ref. [125, 126, 127, 128, 130, 131, 133, 136, 137, 138, 139, 140, 141, 142, 144, 147, 152]).

**Table 2.** **Summary of the key findings from preclinical and clinical studies on currently available transcatheter tricuspid valve replacement devices**.

First author, year	Device name	FDA approved and/or CE-marked	Operational mechanism	Main results	Follow-up
Clinical studies					
Fam, 2021 [126]	EVOQUE	Yes	Orthotopic Valve	↓ TR severity ≤ moderate	30 days
Kodali, 2022 [127]	EVOQUE	Yes	Orthotopic Valve	↓ TR severity ≤ mild	30 days
Kodali, 2023 [128]	EVOQUE	Yes	Orthotopic Valve	↓ TR severity ≤ mild	1 year
				↑ Survival	
				↓ Hospitalization rates	
Hahn, 2025 [130]	EVOQUE	Yes	Orthotopic Valve	↓ TR severity ≤ moderate	1 year
				↑ Quality of life	
Arnold, 2025 [131]	EVOQUE	Yes	Orthotopic Valve	↑ Survival	1 year
				↑ Quality of life	
Hahn, 2019 [133]	GATE	No	Orthotopic Valve	↓ TR severity	30 days
Hahn, 2020 [136]	GATE	No	Orthotopic Valve	↓ TR severity	127 days
				≥ 1 grade	
				↓ Mortality	
Lu, 2020 [137]	LuX-Valve	No	Orthotopic Valve	↓ TR severity ≤ mild	30 days
Mao, 2022 [138]	LuX-Valve	No	Orthotopic Valve	↓ TR severity ≤ mild	1 year
Pan, 2025 [139]	LuX-Valve	No	Orthotopic Valve	↓ TR severity ≤ mild	1 year
Zhang, 2023 [140]	LuX-Valve Plus	No	Orthotopic Valve	↓ TR severity ≤ mild	30 days
Sun, 2025 [141]	LuX-Valve Plus	No	Orthotopic Valve	↓ Intraoperative bleeding	6 months
				Shorter Hospital stay	
Vaturi, 2021 [142]	Trisol	No	Orthotopic Valve	↓ Right ventricular diameter	2 weeks
Teiger, 2022 [144]	Topaz	No	Orthotopic Valve	No residual TR	3 months
				No complications	
Caneiro-Queija, 2023 [147]	CardioValve	No	Orthotopic Valve	No residual TR	3 months
Estévez-Loureiro, 2022 [125]	TricValve	Yes	Heterotopic Valve	↑ Quality of life	6 months
				↓ All-cause mortality	
				↓ HF hospitalizations	
Blasco-Turrión, 2024 [152]	TricValve	Yes	Heterotopic Valve	↑ Quality of life	1 year
				↓ All-cause mortality	

Symbols: ↑ increased; ↓ decreased.

## 4. Conclusions

To date, the progression of TR is often exacerbated by delayed diagnosis and treatment. The limited efficacy of medical therapy and the high procedural risks of surgery make TR a particularly challenging condition to manage. TTVI is an evolving field that aims to offer effective and safe therapeutic options for patients with severe or greater TR who are deemed inoperable. Although preliminary clinical data suggest favorable efficacy and safety for these medical devices, only a limited number have received CE marking and are routinely used in clinical practice. Many clinical trials are still ongoing, while most available studies have very low methodological quality and short follow-up. Therefore, further high-quality, long-term studies are essential to strengthen the evidence for the use of TTVI devices, refine patient selection criteria, and assist cardiologists in identifying the most appropriate and effective devices for individual patients.

## Abbreviations

AFTR, atrial functional tricuspid regurgitation; AV, atrio-ventricular; CAVI, caval valve implantation; CCT, cardiac computed tomography; CCTA, cardiac computed tomography angiography; CE, Conformité Européenne; CIED, cardiac implantable electronic device; CMR, cardiac magnetic resonance; EROA, effective regurgitant orifice area; FDA, U.S. Food and Drug Administration; HF, heart failure; HFpEF, heart failure with preserved ejection fraction; HFrEF, heart failure with reduced ejection fraction; IVC, inferior vena cava; LVEF, left ventricular ejection fraction; MR, mitral regurgitation; PH, pulmonary hypertension; PISA, proximal isovelocity surface area; RHC, right heart catheterization; SGLT-2, sodium-glucose co-transporter-2; SVC, superior vena cava; T-TEER, tricuspid transcatheter edge-to-edge repair; TAPSE/PASP, tricuspid annular plane systolic excursion/pulmonary artery systolic pressure ratio; TR, tricuspid regurgitation; TTE, transthoracic echocardiography; TTV, transcatheter tricuspid valve; TTVI, transcatheter tricuspid valve intervention; TV, tricuspid valve; VFTR, ventricular functional tricuspid regurgitation.
